# Asepsis in Root Canal Operations*Read before the Southern California Dental Association, Los Angeles, May 27, 1918.

**Published:** 1919-04

**Authors:** Elmer S. Best

**Affiliations:** Minneapolis


### ASEPSIS IN ROOT CANAL OPERATIONS *

BY DR. ELMER S. BEST, MINNEAPOLIS

A very definite and easily traced relation often exists between cause and effect. One of the best examples in the practice of dentistry has been brought to light in the work that has been accomplished in connection with periapical or root infections. Those who have investigated it are rather familiar with the effect. We do not have to go out of our way to find it. It surrounds us on every side. We have found myriads of infected areas in connection with pulpless teeth and we have not as yet

* Read before the Southern California Dental Association, Los Angeles, May 27, 1918.

scratched the surface. Occasionally, patients who have no pain in or around their teeth come to the dentist in general practice and ask to have their teeth radiographed, meaning thereby that they wish to place an additional responsibility on his shoulders for the root and gingival conditions. What is going to happen when, say even fifty per cent. of the people who have pulpless teeth decide to have them investigated?

When we are brought to a realization of the magnitude of the condition we are confronted with, we know that there are but two things we can do. We can assume the attitude of the old gentlemen who, upon seeing a camel for the first time, said after a careful survey, "Gosh, there ain't no such animal." Or on the other hand, we can admit that such a condition exists and still adopt one of two positions. First, we can promptly throw up the sponge and admit that the evidence is so overwhelmingly against attempting to do anything with pulpless teeth, that all our efforts will be futile and forthwith adopt an attitude of extreme radicalism; or we can devote some time to careful investigation, letting our conclusions come as a natural sequence.

Strange as it may appear, yet it is a fact that after due consideration of the subject, one becomes impressed with the absence of what he might reasonably expect in the literature as the result of deliberate thinking on this subject, up to about five years ago. The sum total of the conclusions arrived at up to that time, apparently had convinced the majority of our profession that the apical portion of a root was for the purpose of hermetically sealing the root. That it possessed no openings through which infection might be carried or forced into the tissues and that the dental pulp like Topsy in Uncle Tom's Cabin, "Just happened to be, didn't come from nowhere and ain't going nowhere, just grew."

Let us see where a little simple reasoning leads to. First, we examine an extracted tooth. Place a little 10%

collargol in the canal and exert pressure by means of temporary stopping or unvulcanized rubber. Note carefully the number of places the collargol appears on the root surface. We may have to work on several teeth before we become impressed with the significance of this condition. But when we do, we realize that any infection on the inside of the canal hasn't far to travel, and has a great opportunity to get into the surrounding tissues and into the patient's circulation. Does this mean anything to us? If it does, then why do we continue to pour infection into these teeth in the unjustifiable faith that we can control it by means of some drug placed in the root filling material.

There are unquestionably two ways in which infection may reach a root end. It may come via the root canal itself, the most frequently traveled route, or it may arrive through the blood stream. If it comes in this latter manner, why does it select a root end upon which to lodge and develop? This brings us to a consideration of the hematogeneous origin of periapical infection as brought to our attention recently by Ulrich. In the writer's opinion, it must occur about as follows: The action of drugs sealed in the canal passes through the foramina and destroys the peridental membrane. This dead membrane forms an ideal place of incubation for any bacteria that may be passing by. Let us see what some of these drugs are. First of all we will consider arsenic. Probably it has been used more than any other one drug in dentistry. There may be some men who can accurately control the action of this drug in such a manner that, although it will destroy the vitality of the pulp, yet it will cease acting at the root end and confine its activities to the interior of the canal. Some can possibly do it, though I am not in on their secret. One thing is quite certain, however, and that is that the man who during one day's activities sees from ten to twenty-five patients, takes two or three impressions for dentures, inserts a couple of gold foil fillings, prepares four or six cavities for inlays, prepares several teeth for bridge abutments, places a few silicate fillings, attends to an orthodontic appliance and looks after the aches and pains of his various patients in addition to spending a couple of hours in his laboratory and to the time necessary for eating, interviewing patients and visitors, that fellow, mark you, is going to handle a deuce of a problem in trying to edge in enough time to successfully control the action of arsenic. And he is the type of man that makes up the big percentage of our profession. I am intensely interested in that man's problems.

In addition to the use of arsenic we have a vast number of other drugs and combinations of drugs which are placed in root canals temporarily, that is for only a few moments or which are sealed in for days at a time, and those which with a delicate technic are forced through the foramina. Many of these have unquestionably contributed their share toward the destruction of the peridental membrane and the periapical tissues.

Next we have to consider not using the rubber dam. Occasionally, we hear the proud boast, "Why, I haven't used a yard of rubber dam in five and in some cases in twenty-five years." A wonderful boast to be sure, and the interesting part of it is that as a general rule these men claim that their patients do not have trouble with their pulpless teeth. Strange they have not as yet learned the difference between, "Trouble with pulpless teeth," and "Trouble from pulpless teeth." The experience many an unbeliever has had when he has been suddenly and unexpectedly confronted by the ominous insurmountable certainty of danger ahead is nicely depicted in the following literary picture of Sir Douglas Haig, by a London Tommy:

" 'Aig, 'e don't sye much, 'e don't; 'e don't, so to sye, sye nothink; but what 'e don't sye don't mean nothink, not 'arf; and when 'e do sye anythink—my Gawd!"

If you want to take a chance of carrying infection into a canal in the belief that you can get it out, and have explained to your patient the chance you are taking and they are willing, all well and good, go ahead and do not use rubber dam, but not unless you have their consent. Do not try to be clever at someone else's expense. Better use your rubber dam; it may prove a good investment for you and your patient.

Also, do not forget that it is an easy feat to injure and infect the soft tissue of the mouth, so before applying a rubber dam which has been boiled, wash out between the teeth to which the dam is to be applied, with a spray and apply iodin around the necks of the teeth. When the dam has been put in place with a rubber dam holder which can be sterilized, we paint the crowns of the teeth included in the dam with tincture of green soap, distilled water, iodin and alcohol.

At this point we are confronted with a condition which requires some additional study. In the case of a tooth which contains a vital pulp, there will be in all a cavity present containing a large amount of carious dentin and through this cavity we must operate. When shall we remove the decay so as to best fortify ourselves against introducing this infection into the canals? We can leave it until after the dam is applied and in its removal, infect the rubber dam, the clamp and the crowns of the teeth included in the dam. A better plan is to do it after the anesthetic has been injected and has become more effective, when we can thoroughly remove the decay and wash out the cavity before applying the rubber dam. This is probably the better plan.

Next we will consider the use of unsterilized root canal instruments. It is almost inconceivable that for years, dentists, dental surgeons if you please, should have strayed so far from basic principles that they should overlook the danger of plugging a barbed broach into a vital pulp unless that broach had previously been sterilized.

The same thing applies to all other instruments used in root canal operations, and yet you and I know that up to within the past four or five years, sterilization of our fine root canal instruments was not a process in general use. These instruments may be sterilized in various ways, one simple method is by employing the Halverson Sterilizer. Have the water boiling, the instruments on a small tray which comes with the sterilizer are put in the water. From there they are put in the warm air chamber and dried quickly.

Again we may mention another appliance which serves all manner of purposes in the operating room. With it, decay is blown away from infected cavities, pus exuding from pyorrhea pockets is removed, and when it is in about as filthy a condition as the mind of man could conceive, it is called upon on certain occasions to dry out the root canal, forcing its foul, bacteria laden breath far down into the canal. Exit the unclean chip blower that in many cases has never seen even a Saturday night ablution. Let us continue, for to the analytical mind it now becomes very interesting. It appears that we are on the track of some of the things that have given us so much trouble in root end infection.

From day to day we have had on our operating bracket absorbent cotton, which, if sterile to begin with, had within a very short time, due to the atmospheric conditions of the office, and handling, departed very far from its original questionable sanitary condition, and become as contaminated as any instrument would be if left lying around the office for days. To increase the degree of uncleanliness to which this material is subjected it is in small particles removed from the cotton holder and with surgically unclean hands and in many instances microscopically unclean hands, wrapped on bristles and forthwith carefully placed in the root canal.

How many times have we sealed dressings in canals with a material which we now know offered very little protection to the canal and which offered but slight resistance to the egress of bacteria-laden fluids of the mouth? In view of the fact that but very few cements are impervious to moisture, let me ask you, what was passing into that canal from the oral cavity from the time the patient left us until he returned? Does this condition not fairly shock us with possibilities of infection. Recall, if you will, the odor of decomposition that is observed under crowns and bridges set temporarily. Then you know what may go on in a human mouth.

In years gone by how many of us knew, in especially the larger canals, when the apex was reached, and on how many occasions did we force the instruments through the foramina and wound the tissues beyond, creating by such a procedure untold damage and leaving the tissues in a condition of lowered resistance? This is solved by using standardized radiography and radiographing the case before the operation is begun.

In the canals in which we could not gain admittance with the instruments we were, until recently, compelled to use, in how many cases have we destroyed the vitality of the pulp and left it to decompose and set up infection? To further add to the evidence we have accumulated in this work, the following may be of interest. A study of the mesio-buccal and disto-buccal roots of upper molars, both roots of upper first bicuspids, mesial roots of lower molars and lower incisors, in cases pulpless teeth showed over 80% to have infected periapical areas. These roots have the smallest canals of any teeth in the mouth and this condition is so significant that we dare not overlook it.

Have we been careful to dry our canals to the apex in order that we might place the filling where it belonged? We have not if we depended on forcing air into the canals to accomplish the removal of the moisture, be it drug or water. It must be removed with absorbent material. Getting to the end of our list, after we succeeded in removing the pulp tissue, did we really carry the canal filling into the apex? Have we depended upon medicated canal pastes, the strongest point of many of which is their terrific odor? If we have, we failed. And lastly, when we had finished the operation and nicely filled the root, did we seal it tightly with a material which would prevent even moisture from getting at the canal fitting.

Your essayist has tried to present for your consideration what he sees when he reviews the field of root end infection. I am so sure that we can walk right up to this proposition and place our finger on this, and this, and this practice or condition and say, "Eliminate these and you will not have so many periapical infections;" I am so certain of it that I am a confirmed optimist on the subject. There is a great deal of encouragement ahead of us. All over this country men are doing this work the way it should be done, and thank heaven, they are getting wonderful results. As. Dr. Rosenow has said, "It can be done but only you dentists know how difficult it is."—*Pacific Dental Gazette,* March, 1919.

